# Supplementary material for: Epigenetic regulation of spurious transcription initiation in Arabidopsis
Source: Nat Commun. 2020 Jun 26;11:3224. doi: 10.1038/s41467-020-16951-w (PMC7319988; doi:10.1038/s41467-020-16951-w)
Supplement: Supplementary file 1 — Supplementary Information [file 41467_2020_16951_MOESM1_ESM.pdf]

# Supplementary Information for "Epigenetic regulation of spurious transcription initiation in *Arabidopsis*" by Le *et al.*

Le Ngoc Tu[1], Yoshiko Harukawa[1], Saori Miura[1], Damian Boer[2], Akira Kawabe[3], Hidetoshi Saze[*1]
[1] Plant Epigenetics Unit, Okinawa Institute of Science and Technology (OIST), 1919-1 Tancha, Onna-son, Kunigami-gun, Okinawa 904-0495, Japan
[2] Wageningen University & Research, Droevendaalsesteeg 4, 6708 PB Wageningen, Wageningen, Netherlands
[3] Faculty of Life Sciences, Kyoto Sangyo University, Kyoto 603-8555, Japan
∗ E-mail: Corresponding hidetoshi.saze@oist.jp

**a**

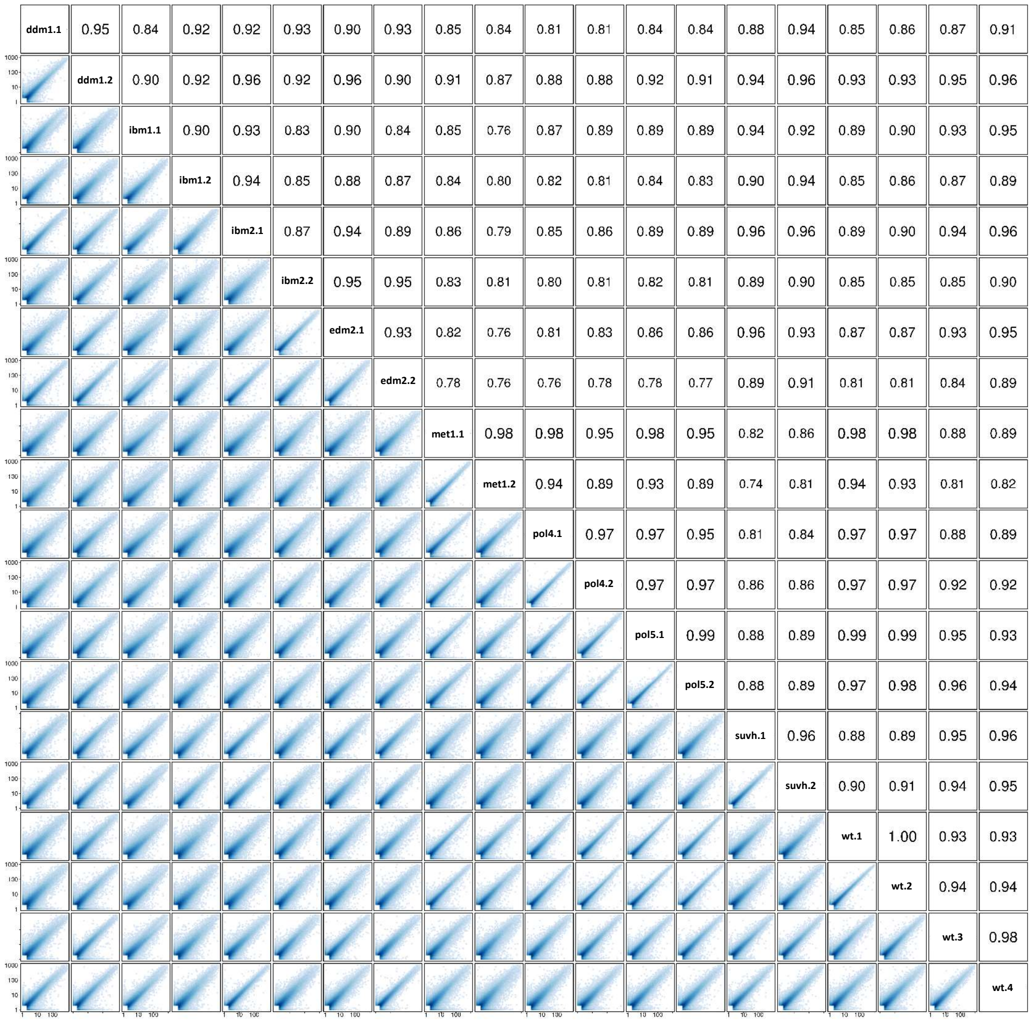


**b**

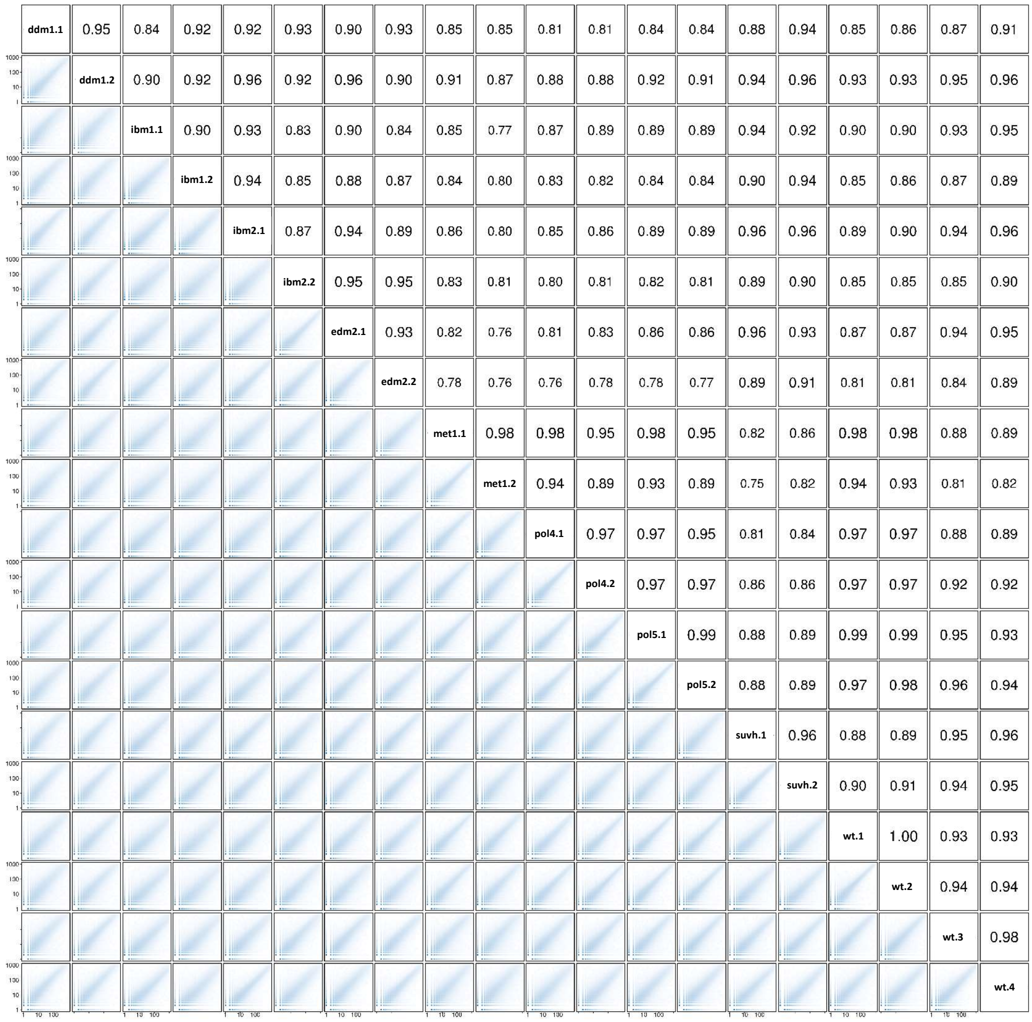


**Supplementary Figure 1.** Reproducibility of CAGE-seq data.

a. Pearson correlation between CAGE-seq samples of the plant *A.thaliana*, calculated based on the normalized expression of individual CTSSs. Sample names are given along the diagonal. On the lower left of the diagonal is the correlation presented in scatter plots.
b. Similar to (a), except that expression was measured by the raw tag counts of individual CTSSs.

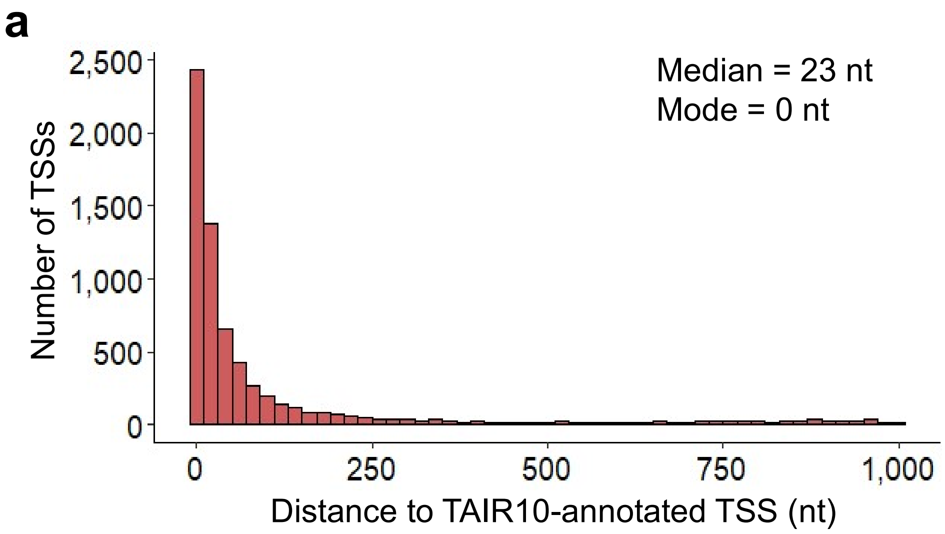


**Supplementary Figure 2.** CAGE-seq data is highly consistent with mRNA-seq and TAIR10 annotation data in identifying TSSs in the *A.thaliana* genome.

a. Histogram of the distances between TSSs identified in promoter regions by CAGE-seq and the annotated TSSs of the same genes in TAIR10.

b. Similar to (b), but TSSs identified in both promoters and 5' UTRs were taken into account. A few TSSs were located in distal 5' UTRs, causing a long tail on the right side of the histogram. The 90th percentile value of this distribution is ~180 nt, which was then used to categorize TSSs into ANNOTATED and NON-ANNOTATED classes shown in Figure 2a.

c. The overlap between active genes identified by CAGE-seq (genes having at least one TSS in promoters or 5' UTRs, blue) and mRNA-seq (genes having at least ten mapped reads across all replicates, red) data. *p=0, Hypergeometric test.

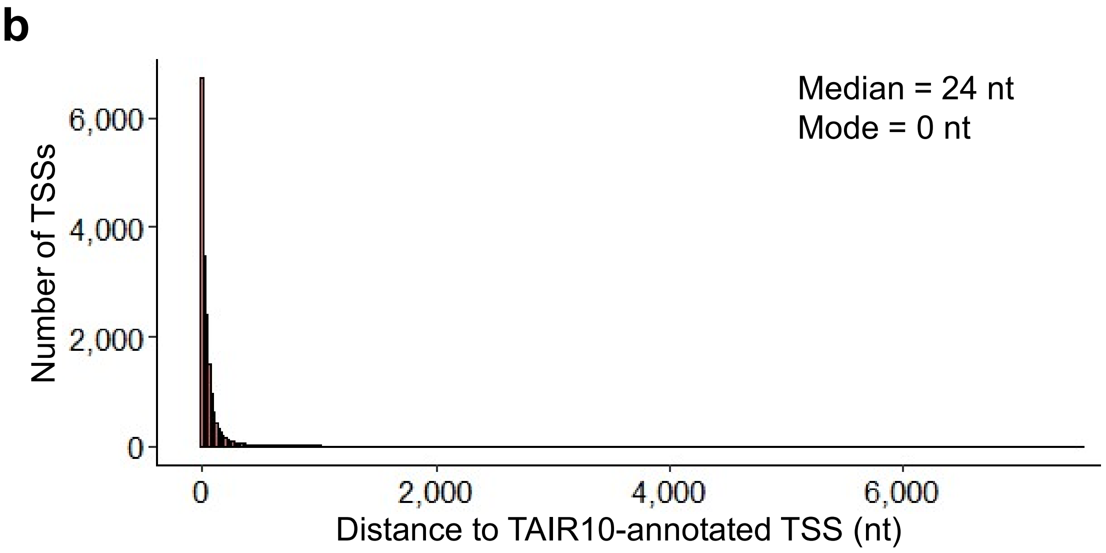


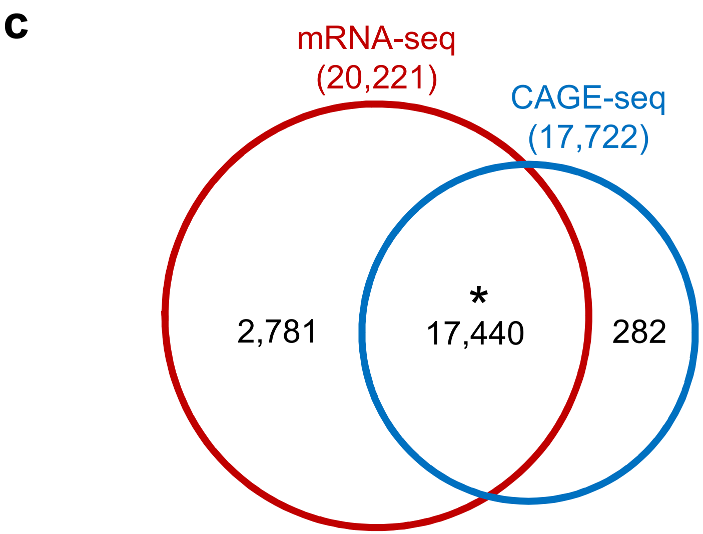

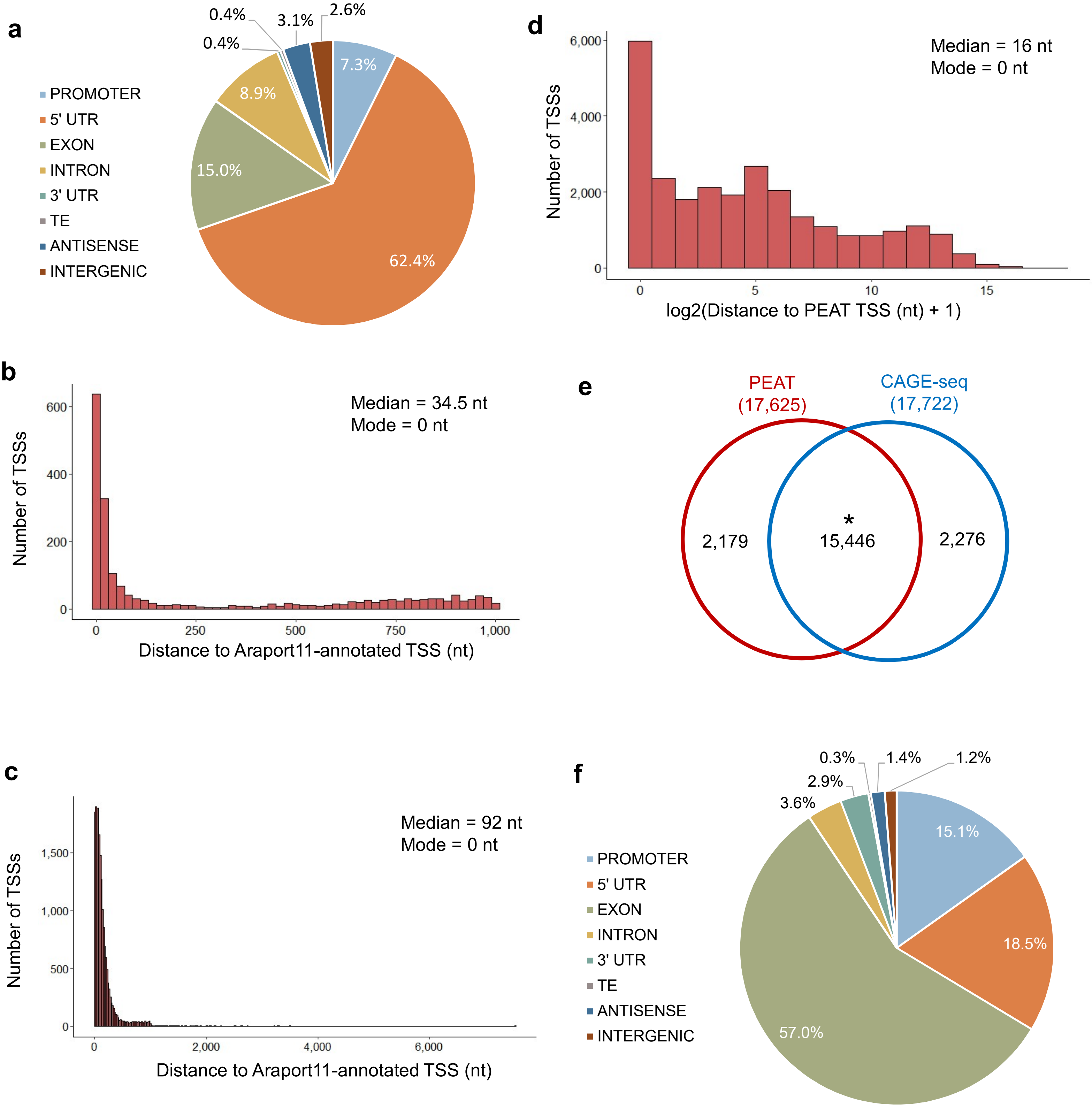


**Supplementary Figure 3.** Consistency of CAGE-seq data with Araport11 annotation and PEAT-seq data.

a. Similar to Figure 1a, Araport11 gene annotations were used instead.

b. Similar to Supplementary Figure 2a, Araport11 gene annotations were used instead.

c. Similar to Supplementary Figure 2b, Araport11 annotations were used instead. The 90th percentile value of this distribution is ~350 nt, which was then used to categorize TSSs into ANNOTATED and NON-ANNOTATED classes shown in Supplementary Figure 5.

d. Histogram of the distances from the TSSs identified by CAGE-seq to the TSSs identified by PEAT-seq (measured by the distance between the dominant TSS of each CAGE-seq tag cluster and the location of the nearest PEAT-seq cluster's mode).

e. Overlap between active genes identified by CAGE-seq and PEAT-seq. *p=0, Hypergeometric test.

f. Genome-wide distribution of TSSs identified by PEAT. Genomic annotations were used as described in Figure 1a. Although about three more times of TSSs (~79,000) were reported by PEAT, a majority of them were found in exons.

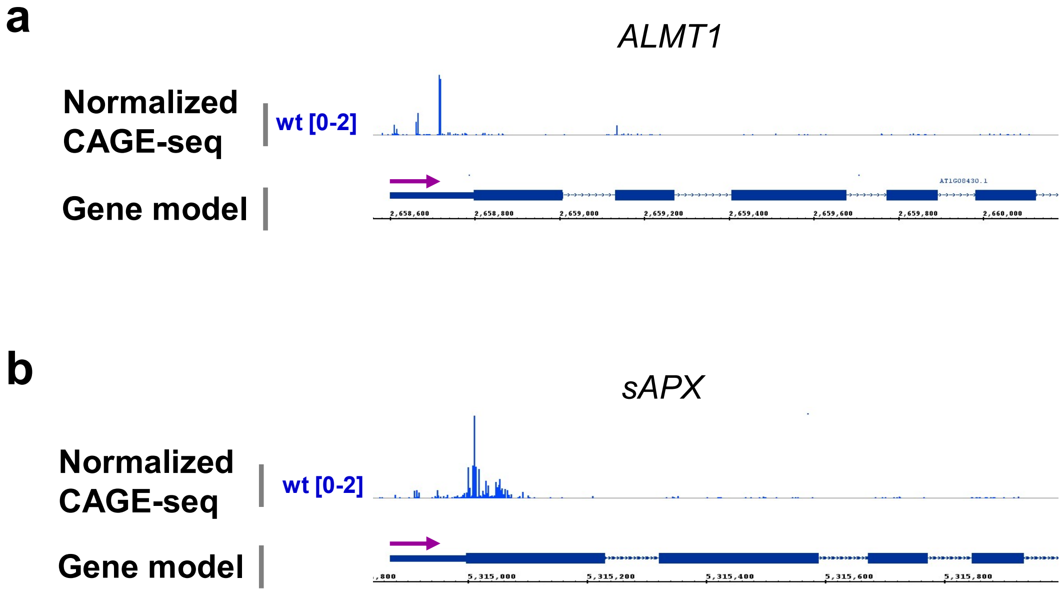


**Supplementary Figure 4.** CAGE-seq data recapitulate the promoter architecture of well-studied genes. Purple arrows indicate the direction of transcription.

a. The promoter architecture of the *ALMT1* (*AT1G08430*) gene identified by CAGE-seq data. The most dominant TSS is located closest to the gene's start codon. Shown is the gene's 5' end.

b. The promoter architecture of the *sAPX* (*AT4G08390*) gene identified by CAGE-seq data. Shown is the 5'-end of a representative isoform of the gene.

**a**

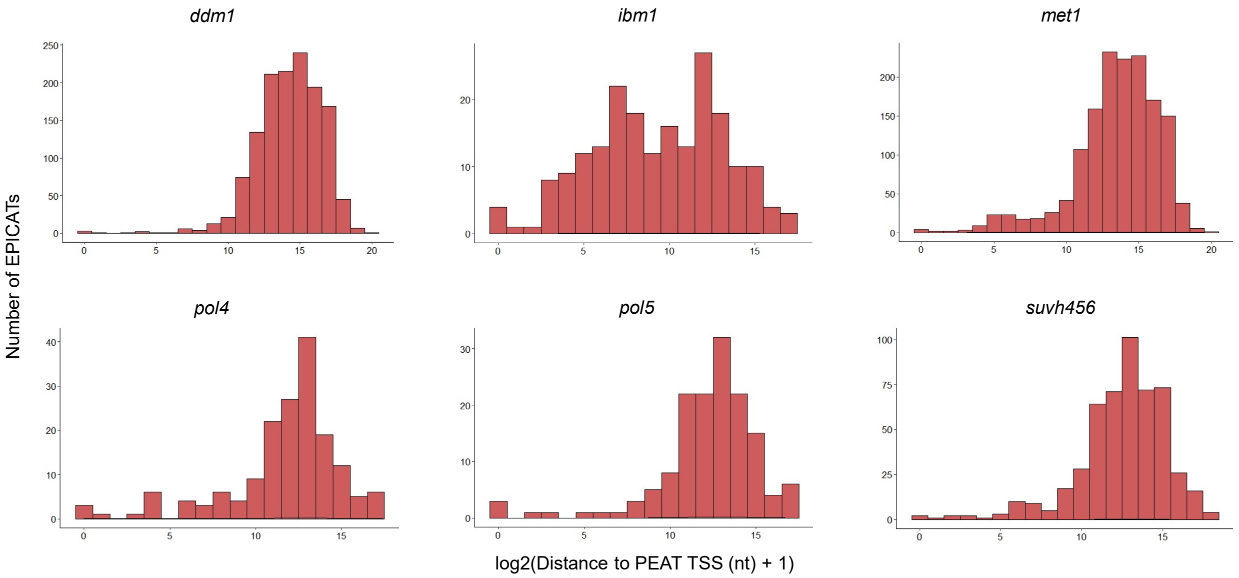


**b**

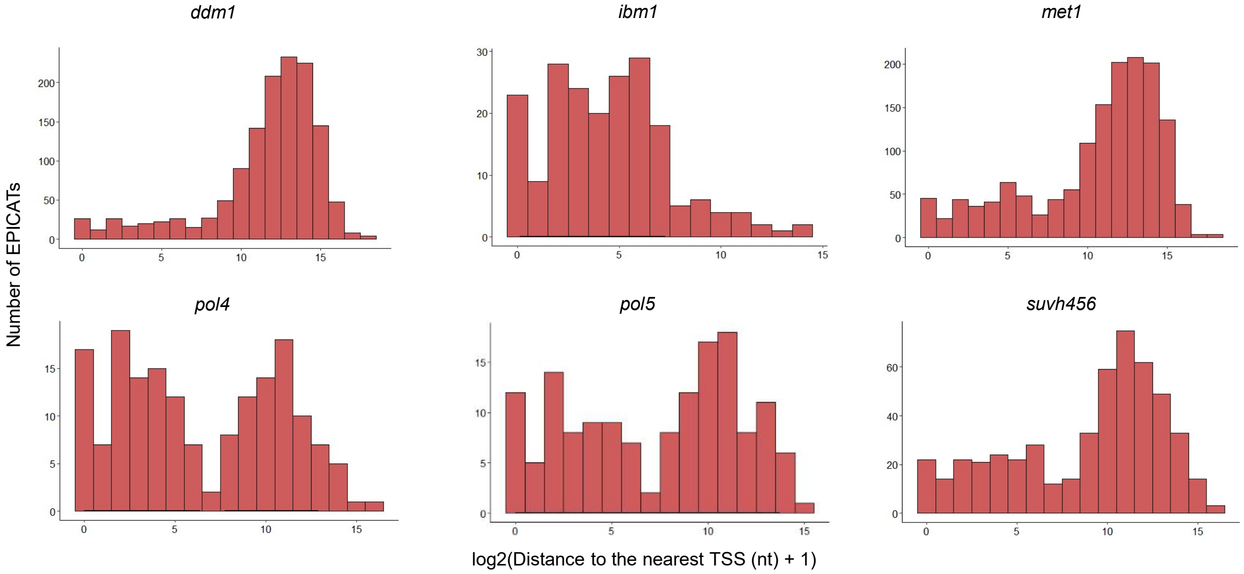


**c**

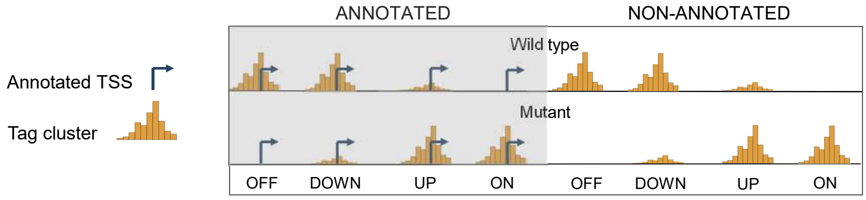


TSSs differentially expressed in epigenetic mutants

| | OFF | DOWN | UP | ON | OFF | DOWN | UP | ON | Total |
|---|---|---|---|---|---|---|---|---|---|
| *ddm1* | 22 | 228 | 294 | 125 | 69 | 84 | 111 | 1294 | **2227** |
| *ibm1* | 121 | 1317 | 1083 | 112 | 360 | 352 | 392 | 178 | **3915** |
| *ibm2* | 8 | 69 | 62 | 6 | 33 | 22 | 41 | 17 | **258** |
| *edm2* | 5 | 312 | 191 | 1 | 22 | 43 | 122 | 10 | **706** |
| *met1* | 103 | 1048 | 803 | 243 | 120 | 257 | 317 | 1411 | **4302** |
| *pol4* | 40 | 470 | 684 | 78 | 42 | 103 | 151 | 154 | **1722** |
| *pol5* | 13 | 109 | 196 | 32 | 8 | 24 | 46 | 134 | **562** |
| *suvh456* | 64 | 578 | 374 | 93 | 116 | 130 | 223 | 475 | **2053** |

Number of tag clusters

0 — 1,500

**EPICATs**

**Supplementary Figure 5.** Distinction of the EPICATs activated in the epigenetic mutants.

a. Histograms of the distances from the EPICATs activated in the epigenetic mutants to the TSSs identified by PEAT-seq (measured by the distance between the dominant TSS of each CAGE-seq tag cluster and the location of the nearest PEAT-seq cluster's mode in the same direction).

b. Histograms of the distances from dominant CTSSs of the EPICATs activated in the epigenetic mutants to the nearest TSSs (in the same direction) identified in multiple tissues and light stress conditions in (Ref. 21).

c. Similar to Figure 2a, except that Araport11 genomic annotations were used and the distance of 350 nt was used to categorize TSSs into ANNOTATED and NON-ANNOTATED classes.

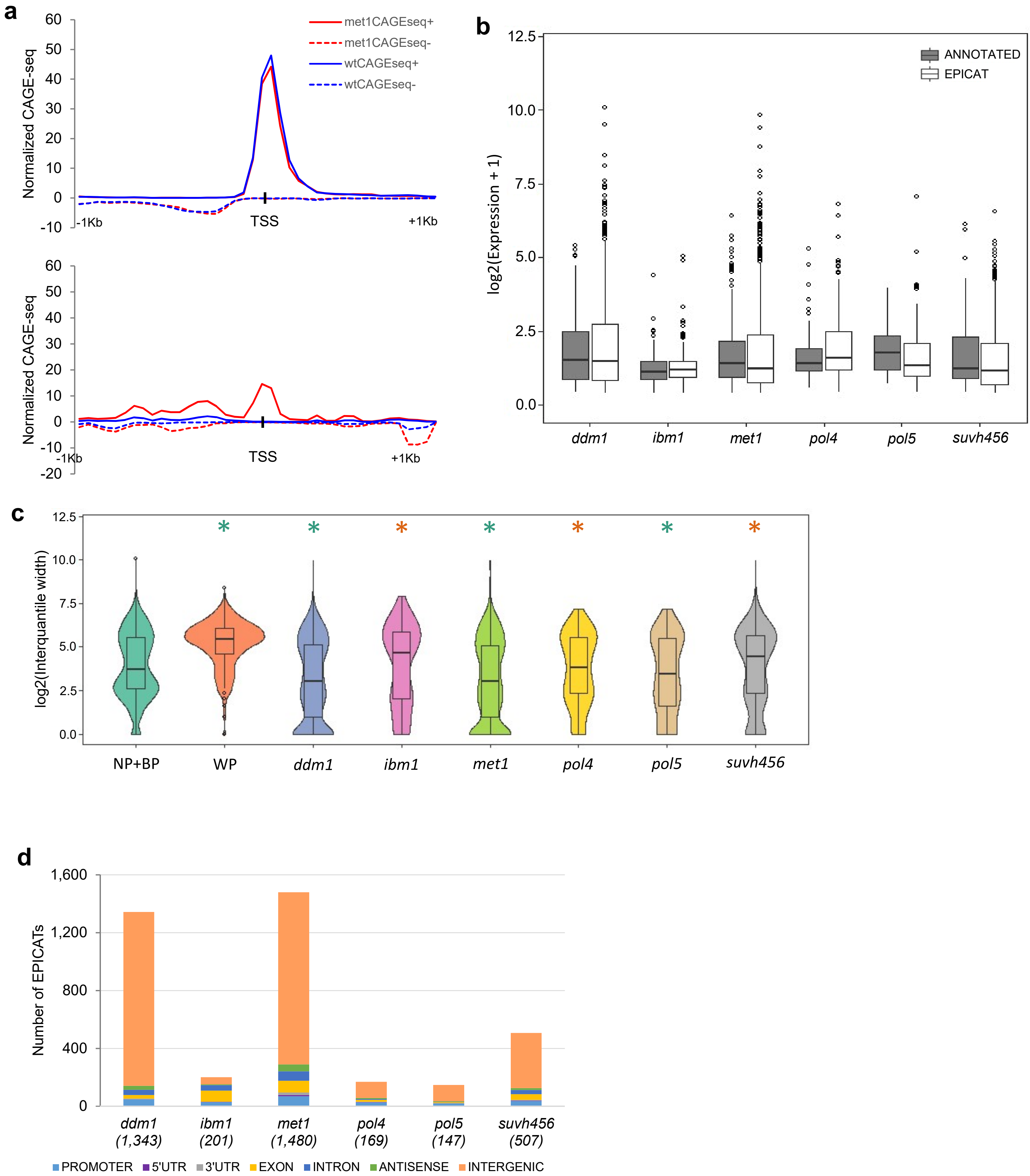


**Supplementary Figure 6**. Features of the transcription at EPICATs.

a. Directionality of transcription at TAIR10-annotated TSSs (upper panel) and the EPICATs in the *met1* mutant (lower panel) of *A. thaliana*. Normalized CAGE-seq signals were calculated for non-overlapping 50 bp windows in a region ±1 Kb around the TSSs (or dominant CTSSs in case of the EPICATs). Shown are their averaged profiles in the *met1* and wild-type (wt) plants.

b. The expressions of EPICATs and *de novo* activated, ANNOTATED TSSs. No significant difference was observed (all p > 0.01 given by two-sided Mann-Whitney test, not shown).

c. Violin plots showing interquantile widths of tag clusters corresponding to the EPICATs activated in the epigenetic mutants. NP+BP, WP: tag clusters corresponding to the wild-type TSSs which exactly matched the Narrow Peak (NP) and Broad with Peak (BP), and Weak Peak (WP) TSSs identified by the PEAT method, respectively (see Methods section for details). *, *: $p < 2.2e-16$, given by two-sided Mann-Whitney test when compared to the widths of the TSSs belonging to NP+BP and WP categories, respectively.

d. Similar to Figure 3a, except the exact numbers of EPICATs activated in the mutant backgrounds are shown.

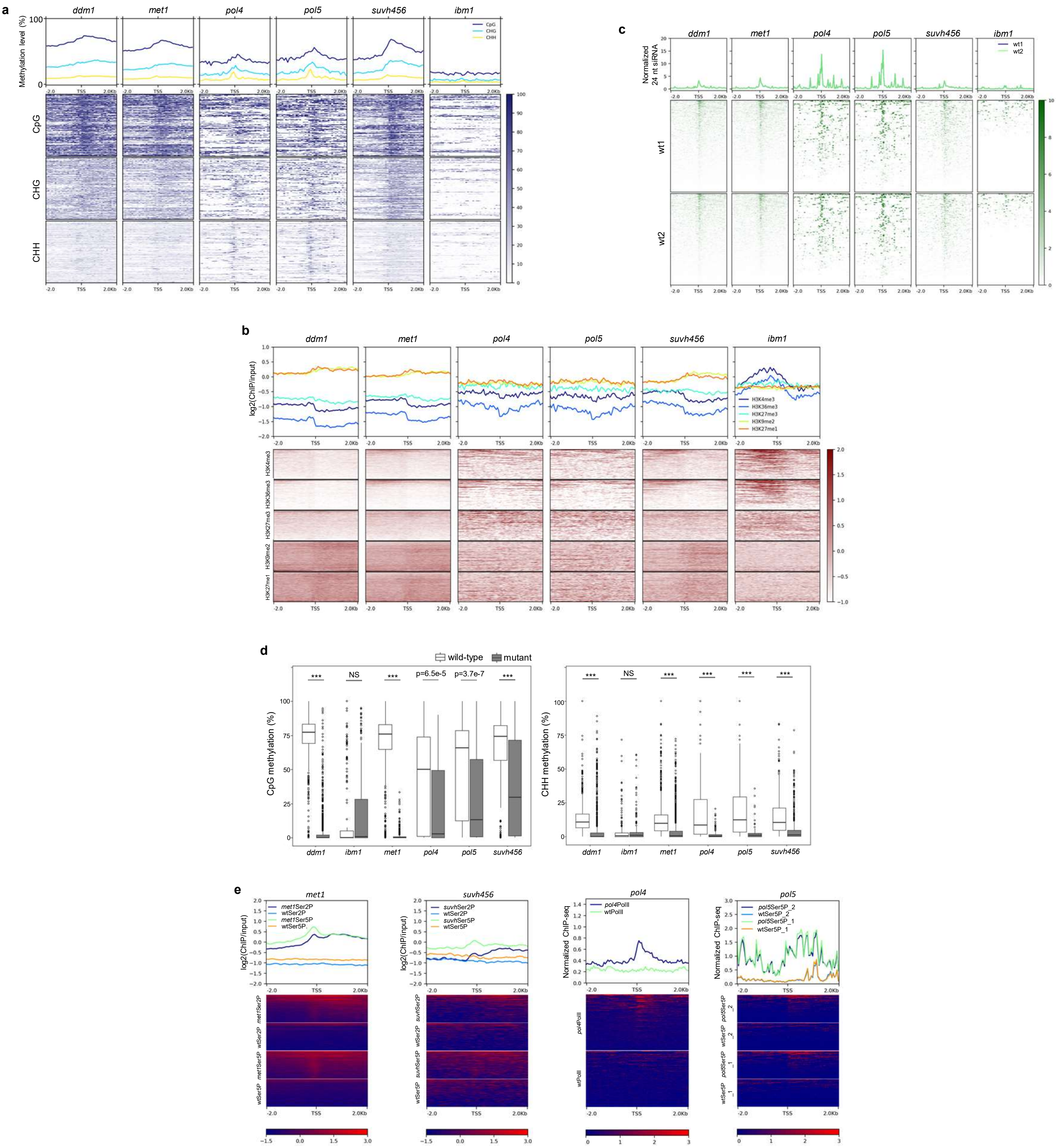


**Supplementary Figure 7.** Features of genomic regions harboring EPICATs.

a. Heatmaps and metaplots showing DNA methylation of genomic regions harboring EPICATs. Methylation levels were calculated for non-overlapping 100 bp windows in the regions of ±2 Kb centering around the EPICATs, aligned by their dominant CTSSs (indicated by TSS), and then sorted by the average value of each row. Columns correspond to the EPICATs activated in each mutant.

b. Heatmaps and metaplots of histone modifications at genomic regions harboring EPICATs. ChIP-seq signals were calculated for non-overlapping 50 bp windows in the regions of ±2 Kb centering around the EPICATs, aligned by their dominant CTSSs (indicated by TSS), and then sorted by the average value of each row. Columns correspond to the EPICATs activated in each mutant.

c. Heatmaps and metaplots showing the accumulation of 24 nt siRNAs in wild-type plants at genomic regions harboring EPICATs. Normalized levels of 24 nt siRNAs were calculated for non-overlapping 100 bp windows in the regions of ±2 Kb centering around the EPICATs, aligned by their dominant CTSSs (indicated by TSS), and then sorted by the average value of each row. Columns correspond to the EPICATs activated in each mutant.

d. Change of DNA methylation in CpG (left) and CHH (right) contexts at the EPICATs in epigenetic mutants compared to wild-type plants. Boxplots and p-values were generated as described in Figure 3b.

e. Heatmaps and metaplots showing the ectopic recruitments of transcription machinery, represented by RNAPII Ser2P and Ser5P, to the EPICATs in *met1* and *suvh456* (denoted by *suvh* in the legend texts), by RNAPII in *pol4*, and by RNAPII Ser5P in two replicates in *pol5*, and corresponding wild-type (wt) backgrounds. ChIP-seq signals were calculated and presented as described in Figure 3d.

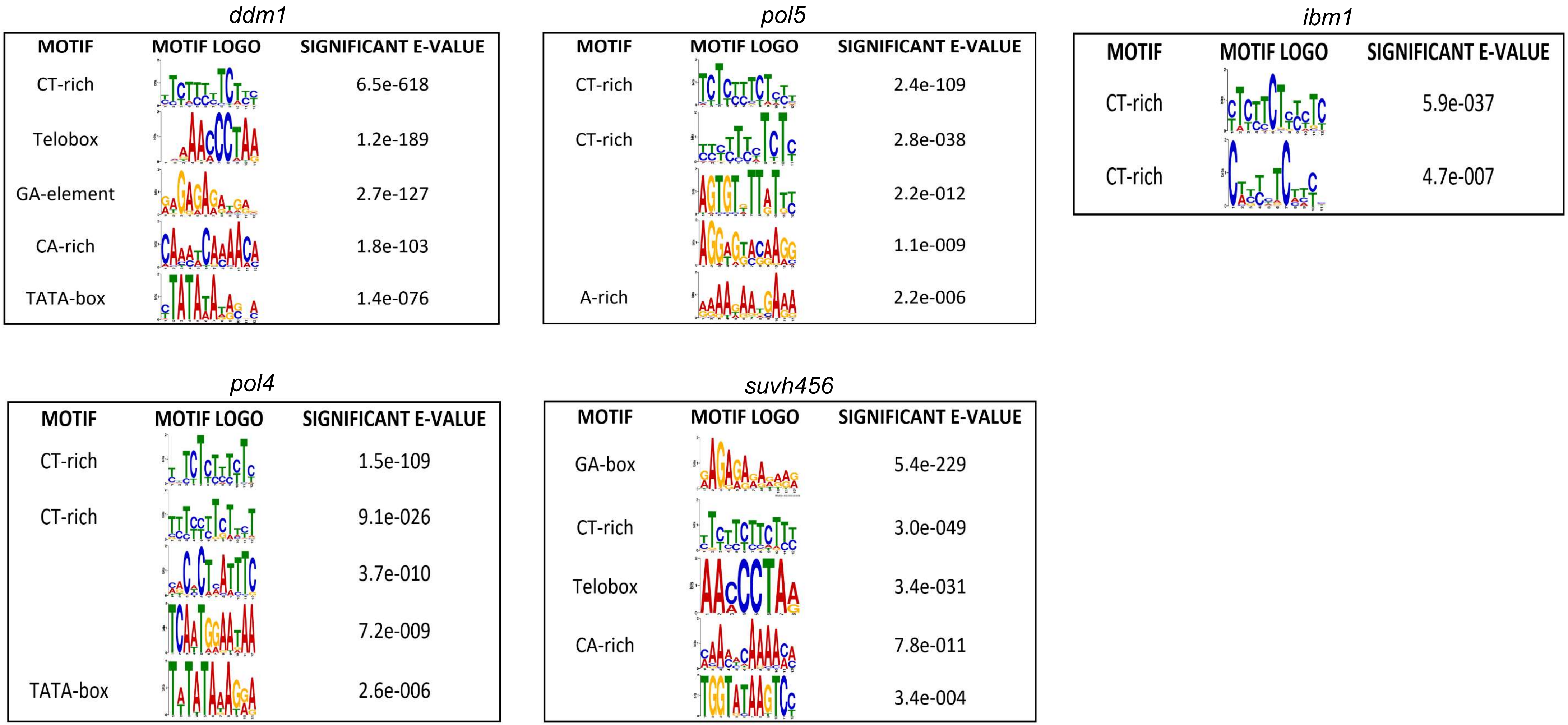


**Supplementary Figure 8.** Top five DNA motifs (or all, if total number of motifs is less than five) significantly enriched (E-value ≤ 0.01) at genomic regions of ±50 bp surrounding dominant CTSSs of the EPICATs activated in *ddm1*, *pol4*, *pol5*, *suvh456,* and *ibm1* given by *de novo* motif analysis. The motifs were selected based on both E-value and the number of sites containing motif instances.

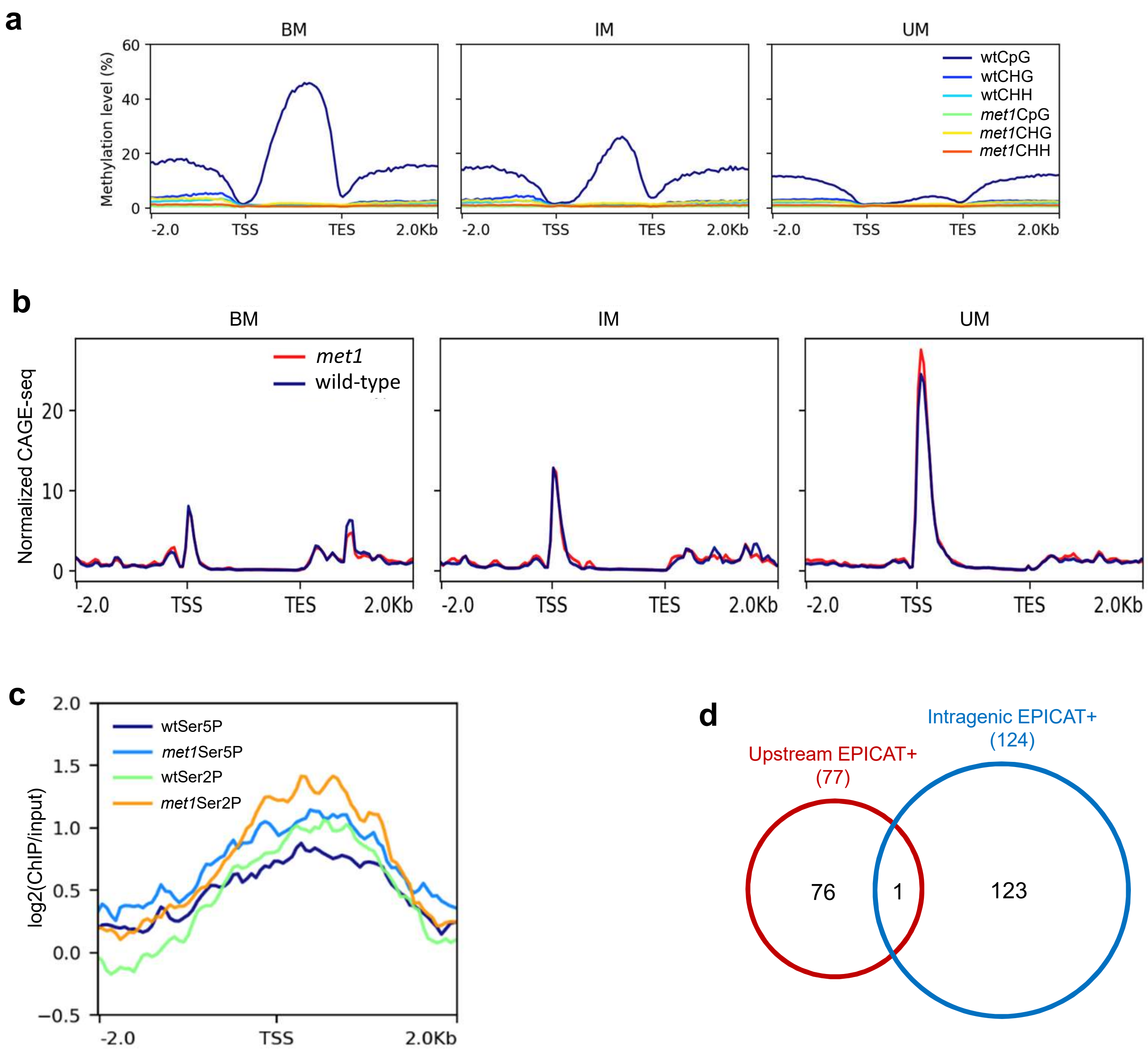


**e**

Consensus motifs at *met1* EPICATs

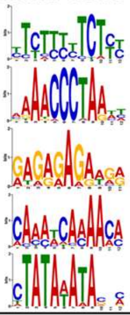

| MOTIF | MOTIF LOGO | SIGNIFICANT E-VALUE | Motif-harboring *intragenic* EPICATs |
|---|---|---|---|
| CT-rich | | 5.3e-657 | 16/125 |
| Telobox | | 4.4e-167 | 1/125 |
| GA-element | | 5.8e-121 | 5/125 |
| CA-rich | | 8.5e-095 | 6/125 |
| TATA-box | | 8.5e-058 | 1/125 |

**Supplementary Figure 9.** The activation of intragenic EPICATs at body methylated genes upon the loss of their gene body methylation (gbM) in *met1.*

a. Metaplots showing the loss of gbM at Body Methylated (BM), Intermediate Methylated (IM), and Unmethylated (UM) genes in *met1*. Gene lengths were normalized to 2 Kb and aligned by their two ends (indicated by TSS and TES, for transcription start and end sites, respectively). DNA methylation were calculated for non-overlapping 50 bp windows within gene body and surrounding regions of ±2 Kb.
b. Metaplots of CAGE-seq signals at the Body Methylated (BM), Intermediate Methylated (IM), and Unmethylated (UM) genes. Normalized CAGE-seq signals were calculated and presented similarly to DNA methylation above.
c. Metaplot showing the distribution of RNAPII Ser5P and Ser2P at the intragenic EPICATs activated in *met1*. ChIP-seq signals were calculated and presented as described in Figure 3d.
d. Overlap between genes containing upstream and intragenic EPICATs in *met1*.
e. Numbers of intragenic EPICATs (right column) harboring consensus motifs enriched at *met1* EPICATs (left panel, Figure 3e).

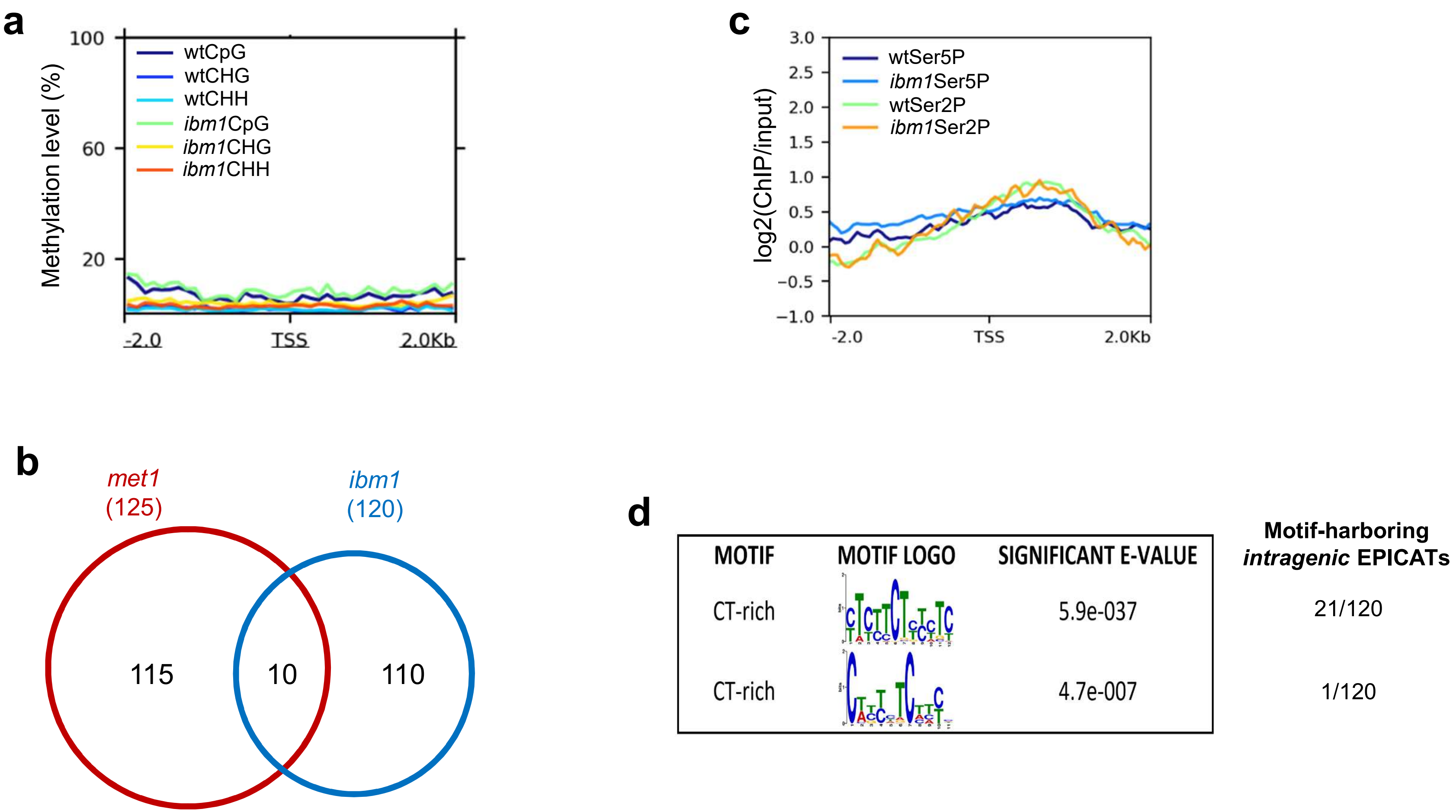


**Supplementary Figure 10.** Features of intragenic EPICATs activated in *ibm1*.

a. Metaplot showing DNA methylation profiles, in wild-type (wt) and *ibm1* backgrounds, at the intragenic EPICATs activated in *ibm1*. The signals were calculated for non-overlapping 50 bp windows in the regions of ±2 Kb centering around the EPICATs and aligned by their dominant CTSSs (indicated by TSS).
b. Overlap between the intragenic EPICATs activated in *met1* and *ibm1*.
c. Metaplot showing the distribution of RNAPII Ser5P and Ser2P at the intragenic EPICATs activated in *ibm1*. Signals were calculated and presented as described in Figure 3d.
d. Numbers of intragenic EPICATs (right column) harboring consensus motifs enriched at all *ibm1* EPICAT loci (left panel, Figure 3e).

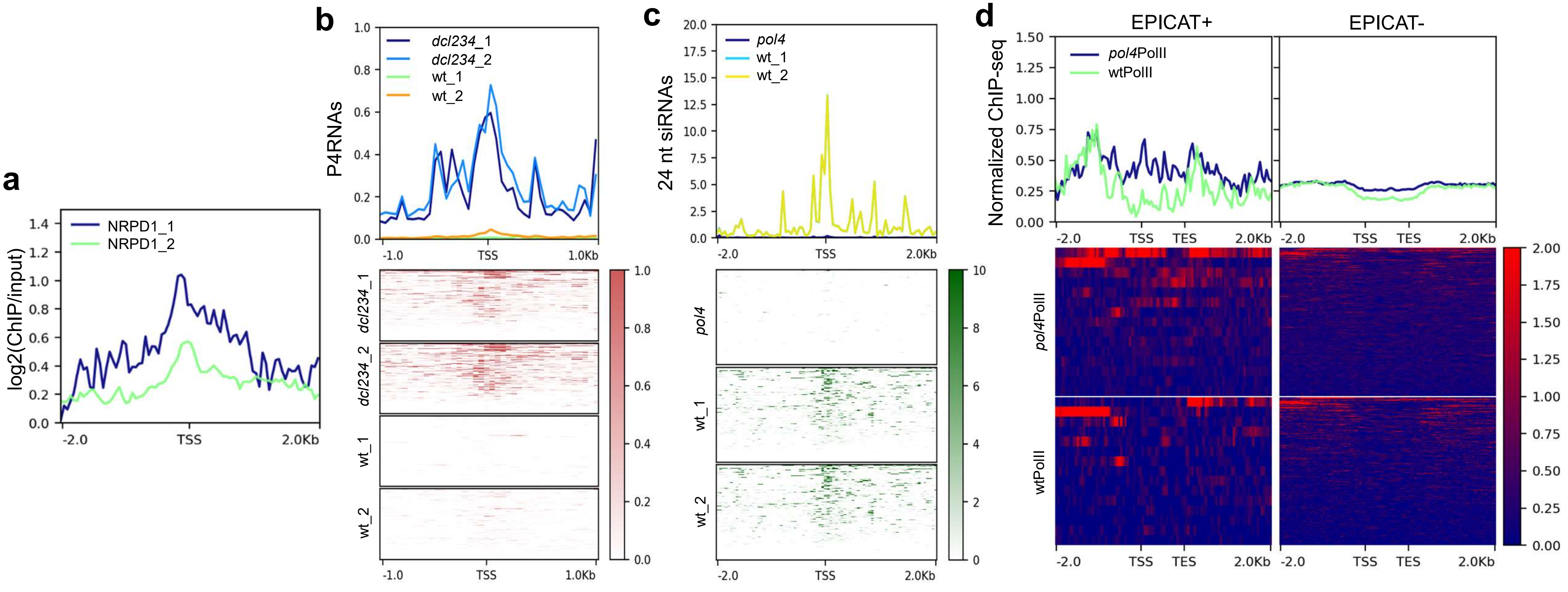


**Supplementary Figure 11.** Competitive binding between RNAPII and PolIV at the EPICATs regulated by the RdDM pathway. All the signals were calculated and presented similarly to RNAPII signals in Figure 3d.

a. Metaplot showing the binding of NRPD1 (two replicates) in wild-type (wt) background at the EPICATs activated in *pol4*.

b. Heatmaps and metaplot showing the accumulation of PolIV-dependent RNAs (P4RNAs) in wild-type (wt) and *dcl2/3/4* backgrounds (two replicates each) at the EPICATs activated in *pol4*.

c. Accumulation of 24 nt siRNAs in wild-type (two replicates) and *pol4* backgrounds at the EPICATs activated in *pol4.*

d. Heatmaps and metaplots showing the binding of RNAPII in wild-type and *pol4* backgrounds at PolIV binding loci, which harbored the activated EPICATs (EPICAT+) or not (EPICAT-).

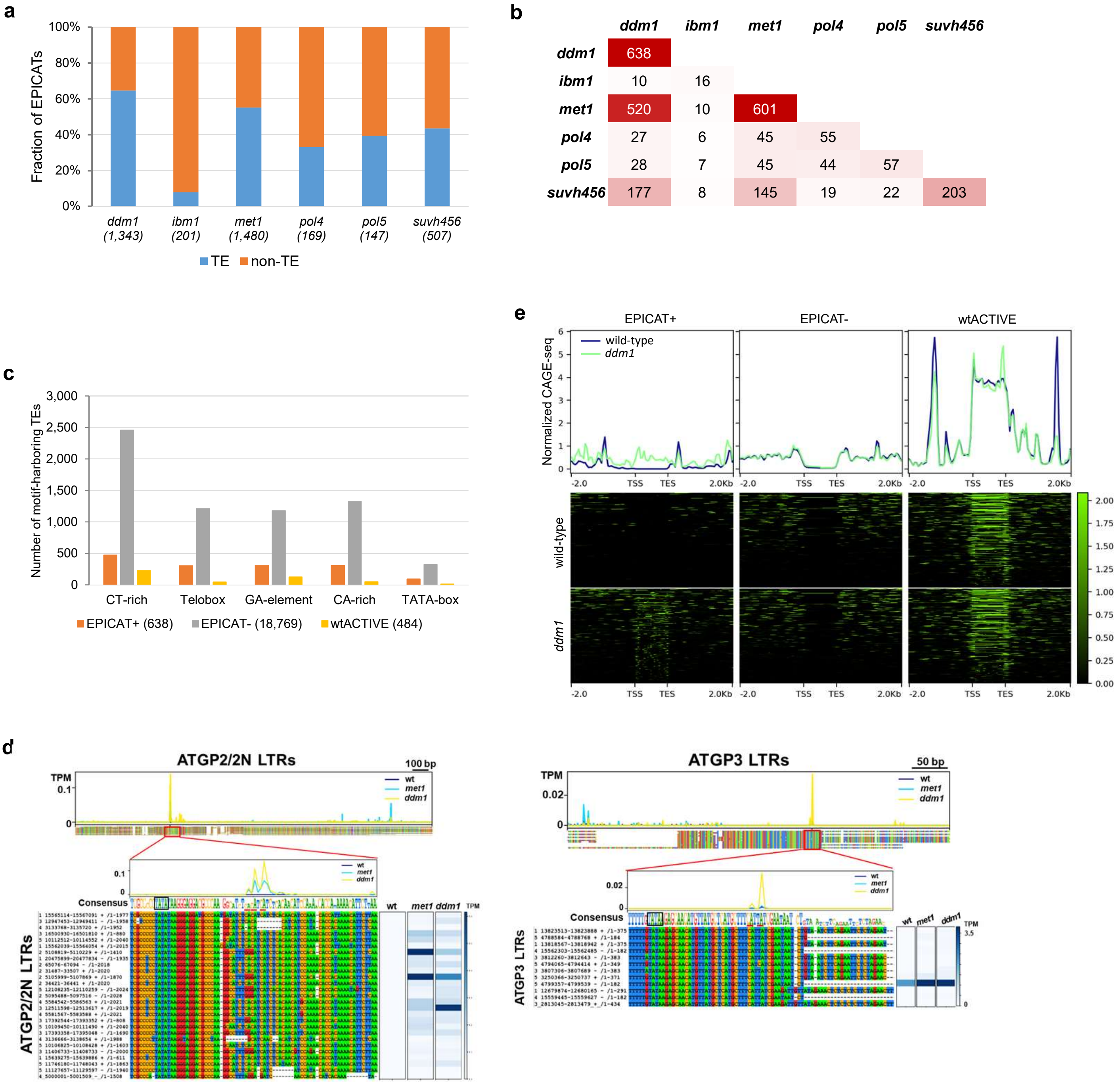


**Supplementary Figure 12.** TEs are a major genetic source of cryptic TSSs in the *A.thaliana* genome.

a. Classification of EPICATs depending on whether they originate from TEs or not (non-TE class). The total number of EPICATs in each mutant is given in the parentheses.
b. The overlaps between TEs harboring the EPICATs in different epigenetic mutants.
c. Similar to Figure 5c, shown are absolute numbers of TE instances.
d. Sequence alignment of representative LTRs of TEs belonging to the Gypsy ATGP2/2N (left) and ATGP3 (right) sub-families. Data are presented as described in Figure 5d.
e. Alteration of transcription initiation at TEs in the *ddm1* mutant compared to the wild-type *A.thaliana*, measured by CAGE-seq data. Normalized CAGE-seq signals were calculated and presented as described in Figure 5e, with the window size of 50 bp.

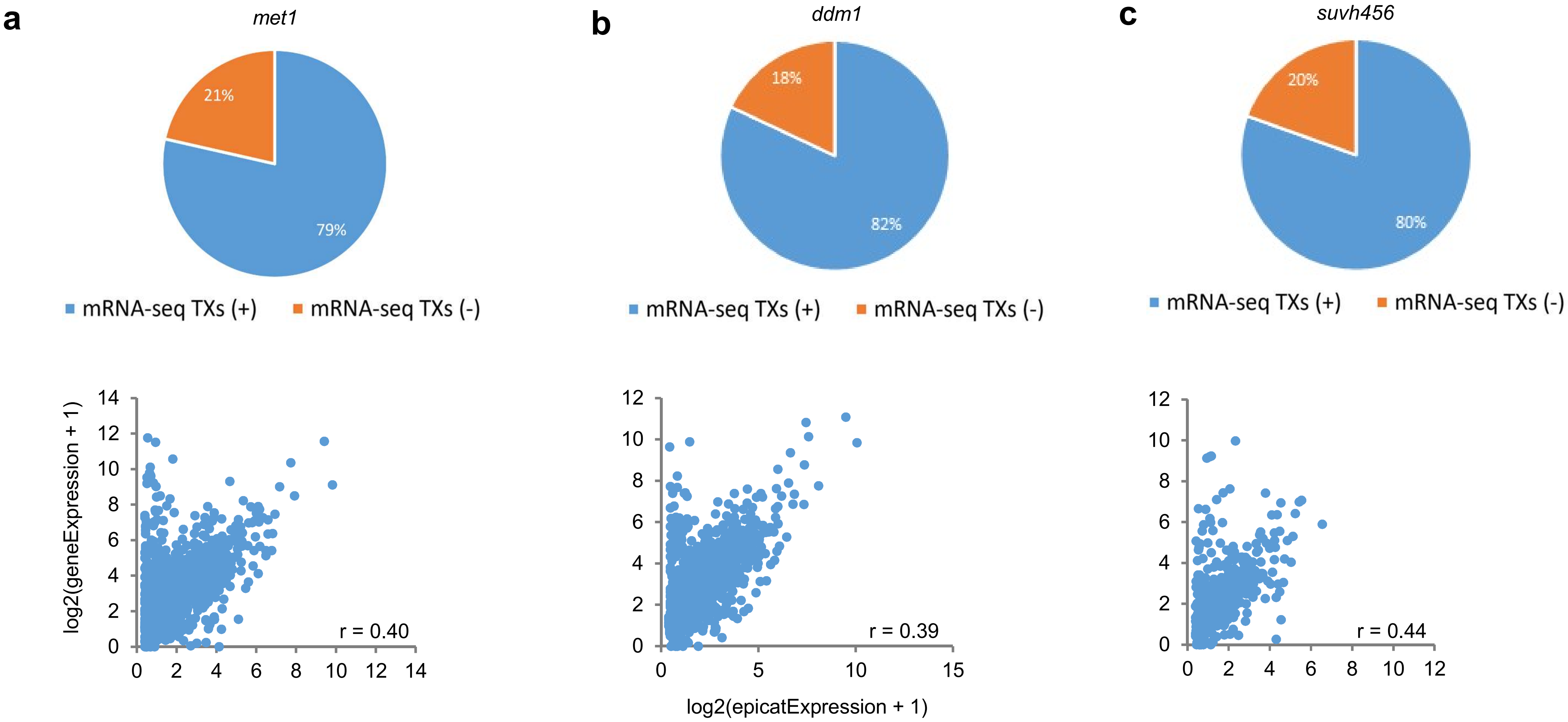


**Supplementary Figure 13.** Relationship between the EPICATs activated in *met1* (a), *ddm1* (b), and *suvh456* (c) and the transcripts assembled from mRNA-seq data. Upper panel: fractions of the EPICATs associated with the assembled transcripts (mRNA-seq TXs+) or not (mRNA-seq TXs-); Lower panel: scatter plots showing the correlations between the expression of the assembled genes and that of the EPICATs. Numbers shown are Pearson correlation coefficients (r). Gene expression was normalized to FPKM (Fragments Per Kilobase Per Million).

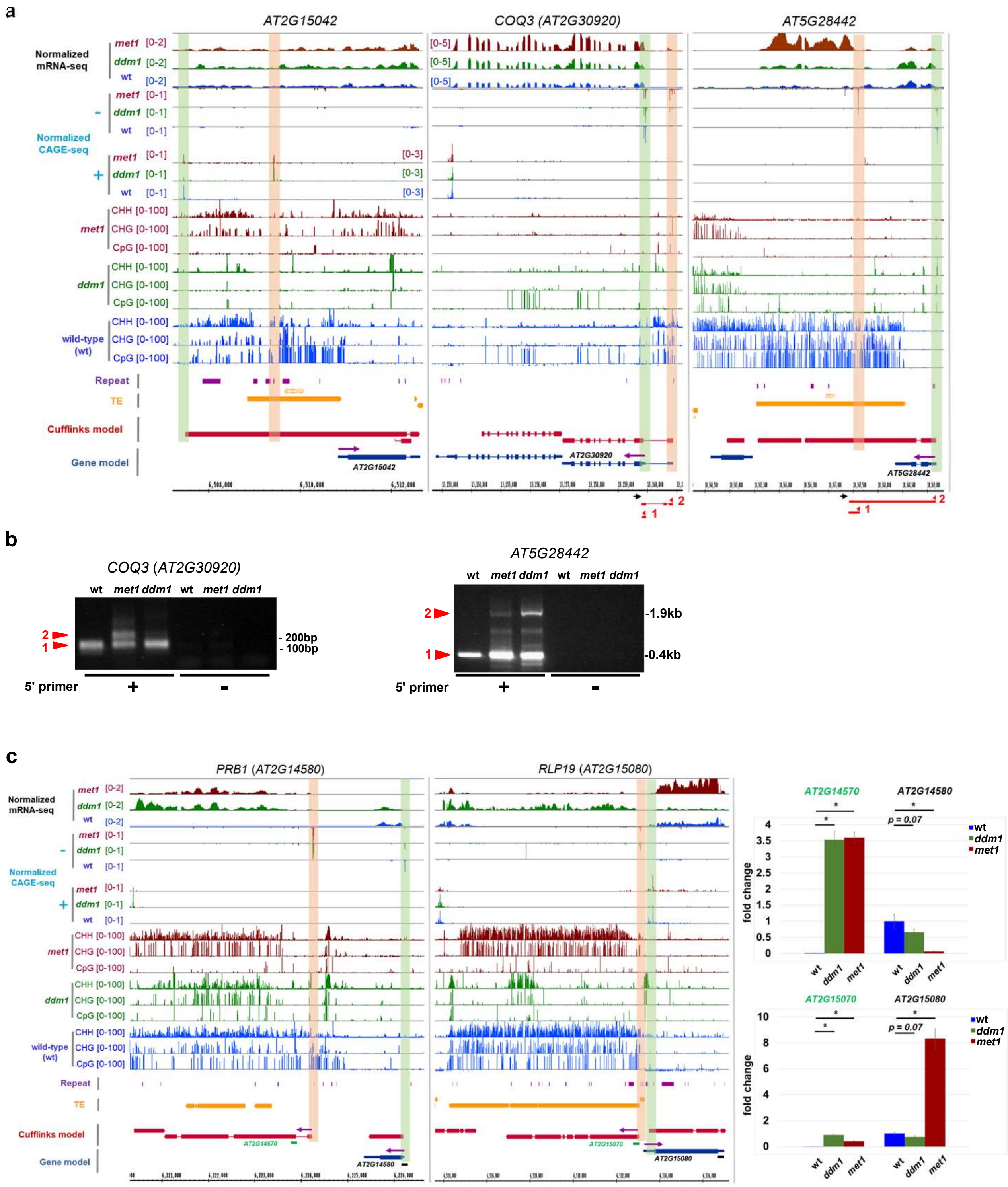


**Supplementary Figure 14.** Transcription alteration caused by the activation of EPICATs in the *Arabidopsis* genome.

a. Browser tracks showing alteration of transcription at *AT2G15042*, *COQ3* (*AT2G30920*), and *AT5G28442* gene loci upon the activation of nearby EPICATs in the *met1* and *ddm1* backgrounds. Data are presented as described in Figure 6b.

b. Detection of cryptic fusion transcripts at the *COQ3 (AT2G30920)* and *AT5G28442* gene loci in the *met1* and *ddm1* backgrounds by 5' RACE. Data are presented as described in Figure 6c, with positions of the detected transcripts indicated by red lines and their 5' ends indicated by red arrows in Supplementary Figure 9a.

c. The attenuation of transcription at the *AT2G14850* and *AT2G15080* gene loci upon the activation of nearby EPICATs in the *met1* and *ddm1* backgrounds. Data are presented as described in Figure 6f.
