# Supplementary material for: Epigenetic regulation of spurious transcription initiation in Arabidopsis
Source: Nat Commun. 2020 Jun 26;11:3224. doi: 10.1038/s41467-020-16951-w (PMC7319988; doi:10.1038/s41467-020-16951-w)
Supplement: Supplementary file 3 — Description of Additional Supplementary Files [file 41467_2020_16951_MOESM3_ESM.pdf]

Descriptions of Additional Supplementary Files

File name: Supplementary Data 1

Description: Summary of CAGE-seq data mapping

File name: Supplementary Data 2

Description: Consensus Tag Clusters (consensusTCs) identified in individual samples and across all samples.

File name: Supplementary Data 3

Description: EPICATs activated in the epigenetic mutants of A.thaliana.

File name: Supplementary Data 4

Description: Gene-body EPICATs in met1 and ibm1. Loci selected for validation by 5'-RACE are marked in red.

File name: Supplementary Data 5

Description: TEs harboring active TSSs in wild-type A.thaliana.

File name: Supplementary Data 6

Description: Transcripts harboring the EPICATs in met1, ddm1, and suvh456.

File name: Supplementary Data 7

Description: Transcripts with significant alternative promoter usage in met1, ddm1, and suvh456. Loci harboring the EPICATs are marked in red, and experimentally validated are marked in blue

File name: Supplementary Data 8

Description: Expression of regular TSSs close to EPICATs. Loci selected for qPCR validation in met1 and ddm1 backgrounds are marked in red. x and y refer to regular TSSs and EPICATs, respectively.

File name: Supplementary Data 9

Description: Summary of ChIP-seq data mapping.

File name: Supplementary Data 10

Description: Positions of LTRs of Gypsy sub-families (ATGPs) analyzed in this study (Figure 5d, Supplementary Figure 12d).

File name: Supplementary Data 11

Description: Primers used in this study.
