# Supplementary material for: Epigenetic regulation of spurious transcription initiation in Arabidopsis
Source: Nat Commun. 2020 Jun 26;11:3224. doi: 10.1038/s41467-020-16951-w (PMC7319988; doi:10.1038/s41467-020-16951-w)
Supplement: Supplementary file 15 — Reporting Summary [file 41467_2020_16951_MOESM15_ESM.pdf]

nature research

Corresponding author(s): Hidetoshi Saze

Last updated by author(s): May 25, 2020

Reporting Summary

Nature Research wishes to improve the reproducibility of the work that we publish. This form provides structure for consistency and transparency in reporting. For further information on Nature Research policies, see Authors & Referees and the Editorial Policy Checklist .

Statistics

For all statistical analyses, confirm that the following items are present in the figure legend, table legend, main text, or Methods section.

n/a	Confirmed	
[ ]	[x]	The exact sample size (*n*) for each experimental group/condition, given as a discrete number and unit of measurement
[ ]	[x]	A statement on whether measurements were taken from distinct samples or whether the same sample was measured repeatedly
[ ]	[x]	The statistical test(s) used AND whether they are one- or two-sided<br>*Only common tests should be described solely by name; describe more complex techniques in the Methods section.*
[x]	[ ]	A description of all covariates tested
[ ]	[x]	A description of any assumptions or corrections, such as tests of normality and adjustment for multiple comparisons
[ ]	[x]	A full description of the statistical parameters including central tendency (e.g. means) or other basic estimates (e.g. regression coefficient) AND variation (e.g. standard deviation) or associated estimates of uncertainty (e.g. confidence intervals)
[ ]	[x]	For null hypothesis testing, the test statistic (e.g. *F*, *t*, *r*) with confidence intervals, effect sizes, degrees of freedom and *P* value noted<br>*Give P values as exact values whenever suitable.*
[x]	[ ]	For Bayesian analysis, information on the choice of priors and Markov chain Monte Carlo settings
[x]	[ ]	For hierarchical and complex designs, identification of the appropriate level for tests and full reporting of outcomes
[ ]	[x]	Estimates of effect sizes (e.g. Cohen's *d*, Pearson's *r*), indicating how they were calculated

Our web collection on statistics for biologists contains articles on many of the points above.

Software and code

Policy information about availability of computer code

Data collection	SRAtoolkit, v2.9.0
Data analysis	FastQC, v0.10.1<br>Trimmomatic, v0.30<br>HISAT2, v2.0.0-beta<br>CAGEr, v1.20.0<br>DESeq2, v1.22.2<br>Rsubread, v1.14.2<br>Cufflinks, v.2.2.1<br>Bowtie, v1.0.0<br>Bowtie2, v2.2.2<br>Bismark, v0.12.1<br>MethylKit, v.0.5.7<br>TrimGalore, v0.4.5<br>Cutadapt, v.1.8.3<br>bedtools, v.2.17.0<br>samtools, v0.1.19<br>MEME, v4.11.2<br>LTR_FINDER, v1.0.2<br>BLAST, v2.0<br>ClustalW, v2.1<br>Jalview, v2.11.0<br>deepTools, v3.3.0

IGB, v.9.1.2
ggplot2,v2.3.1
R, v3.1.0

For manuscripts utilizing custom algorithms or software that are central to the research but not yet described in published literature, software must be made available to editors/reviewers. We strongly encourage code deposition in a community repository (e.g. GitHub). See the Nature Research guidelines for submitting code & software for further information.

Data

Policy information about availability of data

All manuscripts must include a data availability statement. This statement should provide the following information, where applicable:
- Accession codes, unique identifiers, or web links for publicly available datasets
- A list of figures that have associated raw data
- A description of any restrictions on data availability

Sequencing data generated in this study have been deposited to the DDBJ Sequence Read Archive under the accession codes DRA009134 and DRA009847. Their identifications are described in the Methods section. Processed CAGE-seq data are also accessible via the following web link: https://plantepigenetics.oist.jp/.

Field-specific reporting

Please select the one below that is the best fit for your research. If you are not sure, read the appropriate sections before making your selection.

- [x] Life sciences
- [ ] Behavioural & social sciences
- [ ] Ecological, evolutionary & environmental sciences

For a reference copy of the document with all sections, see nature.com/documents/nr-reporting-summary-flat.pdf

Life sciences study design

All studies must disclose on these points even when the disclosure is negative.

Sample size	No sample size calculation was performed. Sample size was chosen according to the standard in the plant biology field.
Data exclusions	One replicate of met1 CAGE samples was excluded from the analyses in the revised manuscript due to a low correlation to the other replicates and according to the reviewers' comments. Another met1 CAGE replicate was discarded due to low mapping coverage.
Replication	All the experiments were reproducible in the repeated experiments.
Randomization	Plant samples were placed randomly in the plant facility.
Blinding	No blinding was applied for sampling. Since most of the data were obtained by bioinformatic analysis with identical parameter settings in our study, blind sampling was not essential.

Reporting for specific materials, systems and methods

We require information from authors about some types of materials, experimental systems and methods used in many studies. Here, indicate whether each material, system or method listed is relevant to your study. If you are not sure if a list item applies to your research, read the appropriate section before selecting a response.

Materials & experimental systems

n/a	Involved in the study	
[ ]	[x]	Antibodies
[x]	[ ]	Eukaryotic cell lines
[x]	[ ]	Palaeontology
[x]	[ ]	Animals and other organisms
[x]	[ ]	Human research participants
[x]	[ ]	Clinical data

Methods

n/a	Involved in the study	
[ ]	[x]	ChIP-seq
[x]	[ ]	Flow cytometry
[x]	[ ]	MRI-based neuroimaging

Antibodies

Antibodies used	Anti-RNA polymerase II CTD repeat YSPTSPS (phospho S2) (Abcam ab5095); Anti-RNA polymerase II CTD repeat YSPTSPS (phospho S5) (Abcam ab5408).
Validation	Validation of the antibodies were available on the manufacture's web site: Abcam ab5095 (https://www.abcam.co.jp/rna-polymerase-ii-ctd-repeat-ysptsps-phospho-s2-antibody-chip-grade-ab5095.html). Abcam ab5408) (https://www.abcam.co.jp/

rna-polymerase-ii-ctd-repeat-ysptsps-phospho-s5-antibody-4h8-chip-grade-ab5408.html).

ChIP-seq

Data deposition

- [x] Confirm that both raw and final processed data have been deposited in a public database such as GEO.
- [ ] Confirm that you have deposited or provided access to graph files (e.g. BED files) for the called peaks.

Data access links<br>*May remain private before publication.*	All the data have been deposited to the DDBJ Sequence Read Archive (accession numbers: DRA009134 and DRA009847).
Files in database submission	A list of files in database submission is provided in the Supplementary Data 9.
Genome browser session<br>(e.g. UCSC)	N/A

Methodology

Replicates	Two replicates for each experiment were performed.
Sequencing depth	Sequencing depths are provided in the Supplementary Data 9.
Antibodies	Anti-RNA polymerase II CTD repeat YSPTSPS (phospho S2) (Abcam ab5095); Anti-RNA polymerase II CTD repeat YSPTSPS (phospho S5) (Abcam ab5408)
Peak calling parameters	No peak calling was performed. ChIP-seq data were used to visualize the accumulation of RNAPII at specific genomic regions.
Data quality	Data quality was assessed by FastQC, v0.10.1.
Software	Trimmomatic, v0.30<br>Bowtie, v1.0.0<br>samtools, v0.1.19
